# Supplementary material for: The missing linker between SUN5 and PMFBP1 in sperm head-tail coupling apparatus
Source: Nat Commun. 2021 Aug 13;12:4926. doi: 10.1038/s41467-021-25227-w (PMC8363609; doi:10.1038/s41467-021-25227-w)
Supplement: Supplementary file 1 — Supplementary Information [file 41467_2021_25227_MOESM1_ESM.pdf]

Supplementary Information

The missing linker between SUN5 and PMFBP1 in sperm head-tail coupling apparatus

Ying Zhang[1#], Chao Liu[2,3#], Bingbing Wu[3,4], Liansheng Li[1], Wei Li[2,3,4*], Li Yuan[1*]

This PDF file includes:

Supplementary Figures 1-7

Supplementary Table 1

Supplementary Figures

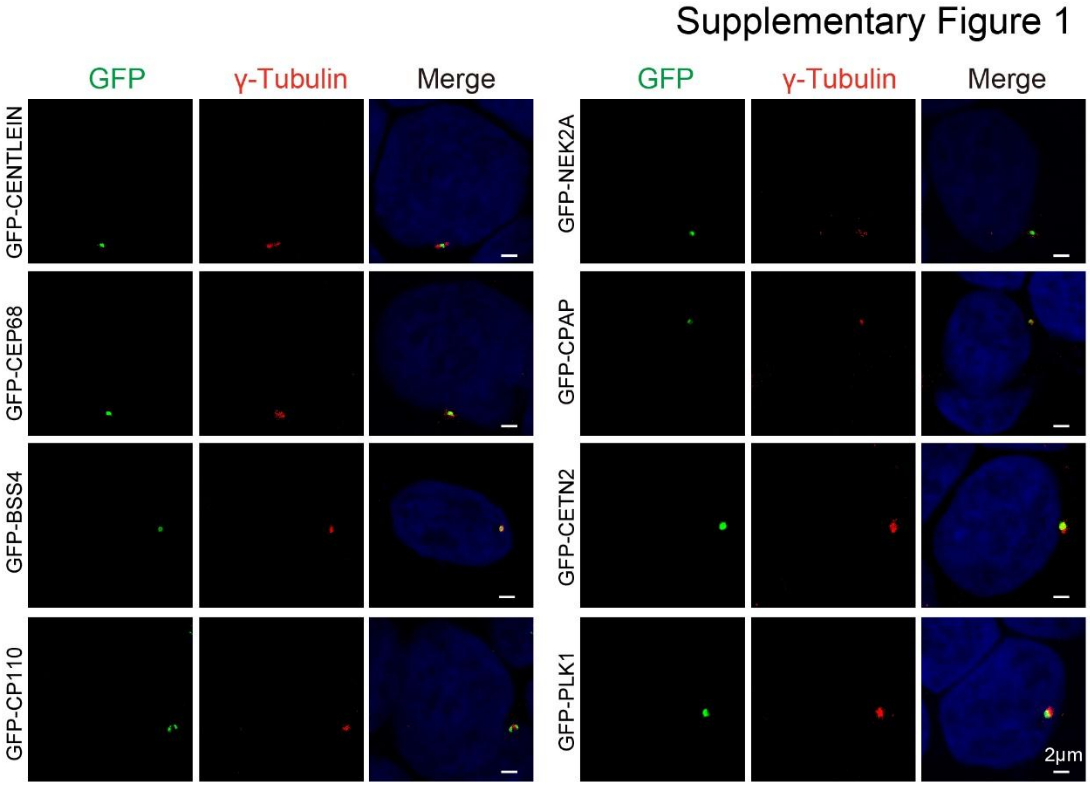

Supplementary Figure1. The immunofluorescence analysis for GFP-tagged proteins (green) in Figure 1a and γ-Tubulin (red) was performed in HEK293T cells. Nuclei were stained with DAPI (blue). The experiment was repeated three times independently with similar results.

Supplementary Figure 2

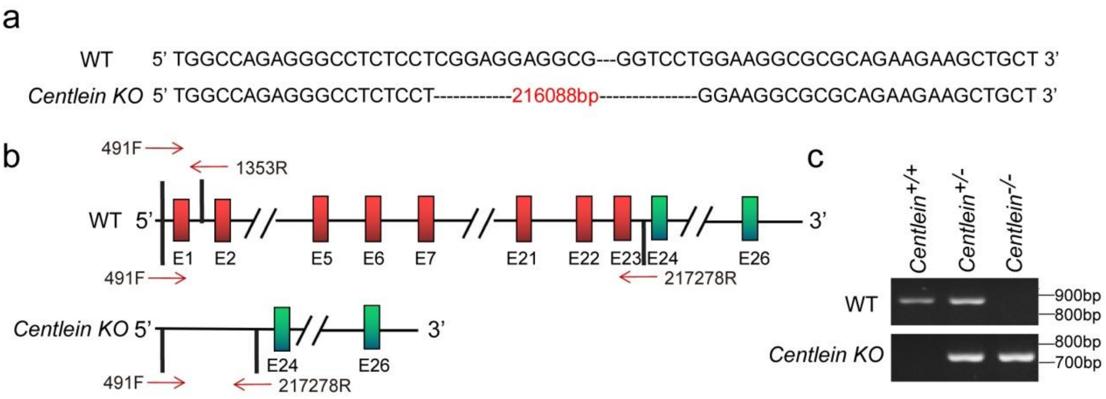

Supplementary Figure 2. The generation of *Centlein* knockout mice. a, Sequences of the WT and *Centlein* mutant alleles in mice. **b,c**, Genotyping of founders to identify *Centlein* knockouts. The gel images were taken immediately using UVP GelDoc-It 310 imaging system with TS2 software. Biologically independent mice were examined in three separate experiments with similar results.

Supplementary Figure 3

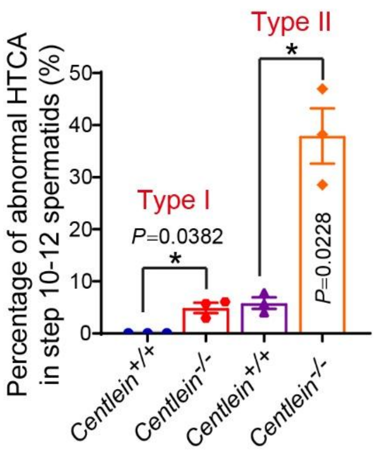

Supplementary Figure 3. Quantitative analysis of the two types of defective HTCA ultrastructure in *Centlein*$^{+/+}$ and *Centlein*$^{-/-}$ step 10-12 spermatids (n=3 independent experiments). Data are presented as mean ± SEM. A two-tailed Student’s t test was performed, *$P < 0.05$. Source data are provided as a Source Data file.

Supplementary Figure 4

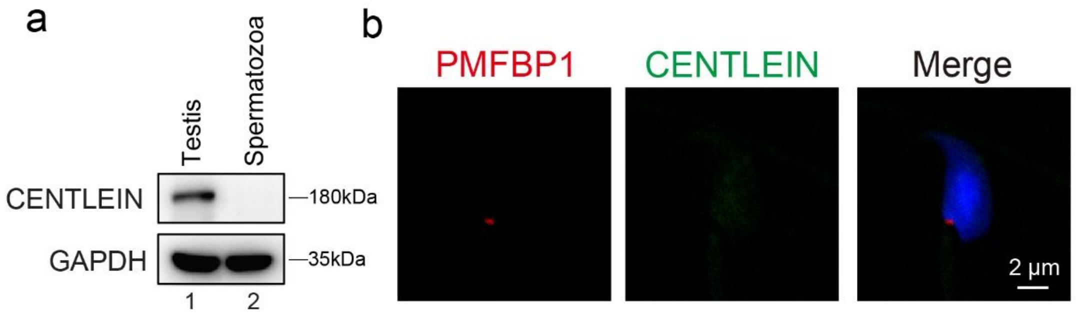

Supplementary Figure 4. CENTLEIN is absence in mature spermatozoa in caudal epididymis. a, Immunoblotting of CENTLEIN was performed in testis and mature spermatozoa from caudal epididymis. GAPDH served as the loading control. The experiment was repeated three times independently with similar results. **b**, The immunofluorescence analysis CENTLEIN (green) and PMFBP1 (red) was performed in mature spermatozoa from caudal epididymis. Nuclei were stained with DAPI (blue). The experiment was repeated three times independently with similar results.

Supplementary Figure 5

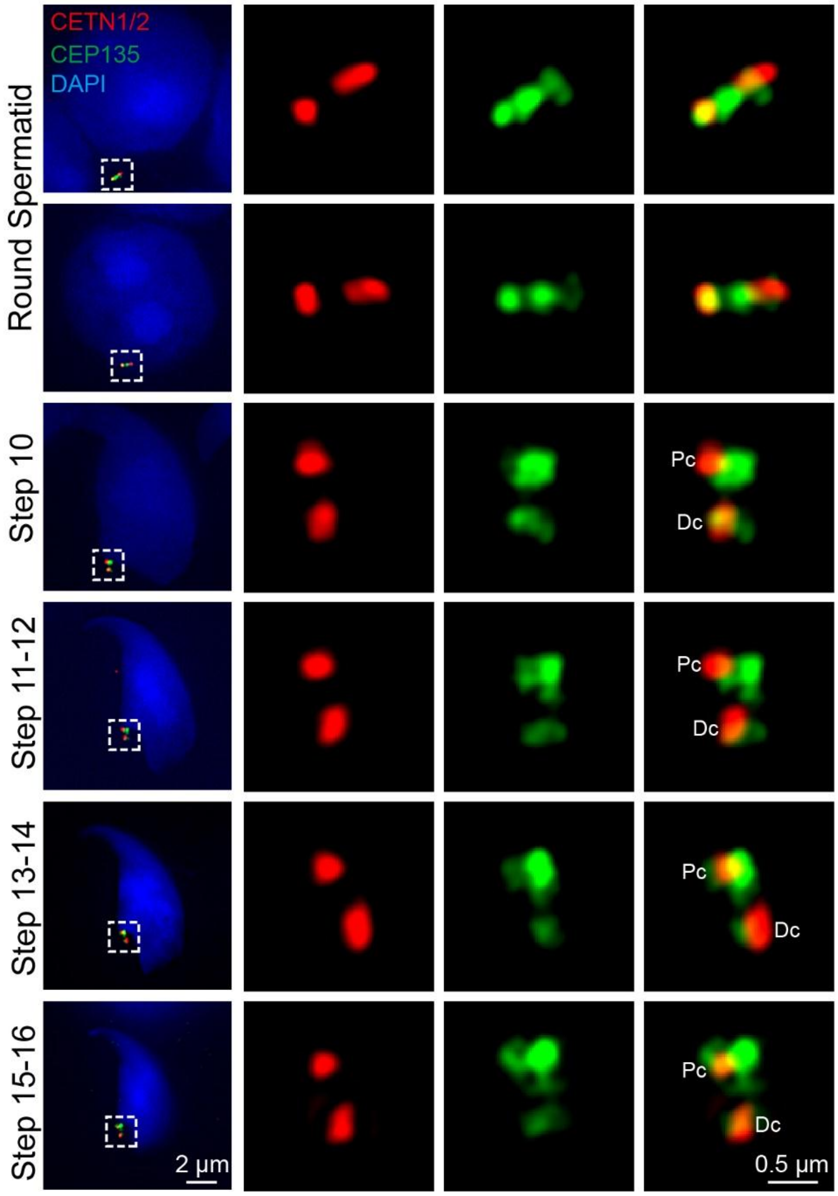

Supplementary Figure 5. Super-Resolution microscopy images of CEP135 (green) and CETN1/2(red) in testicular germ cells. Nuclei were stained with DAPI (blue). Pc: proximal centriole, Dc: distal centriole. The experiment was repeated three times independently with similar results.

Supplementary Figure 6

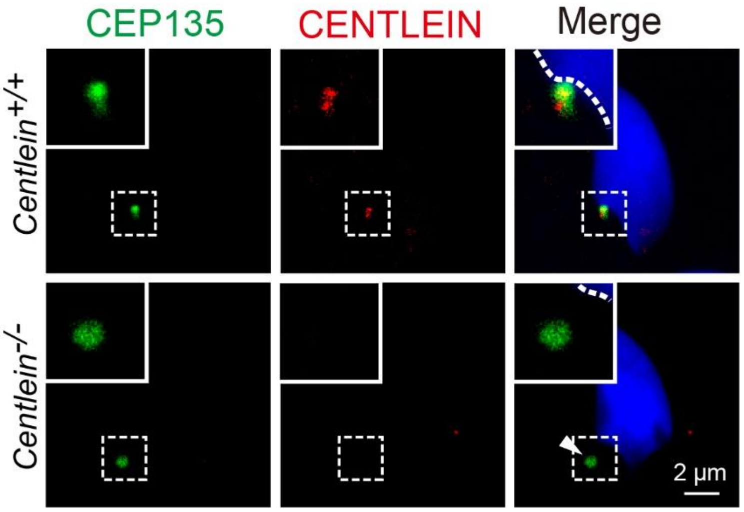

Supplementary Figure 6. Immunofluorescence analysis for CEP135 (green) and CENTLEIN (red) was performed in *Centlein*$^{+/+}$ and *Centlein*$^{-/-}$ testicular germ cells. Nuclei were stained with DAPI (blue). The experiment was repeated three times independently with similar results.

Supplementary Figure 7

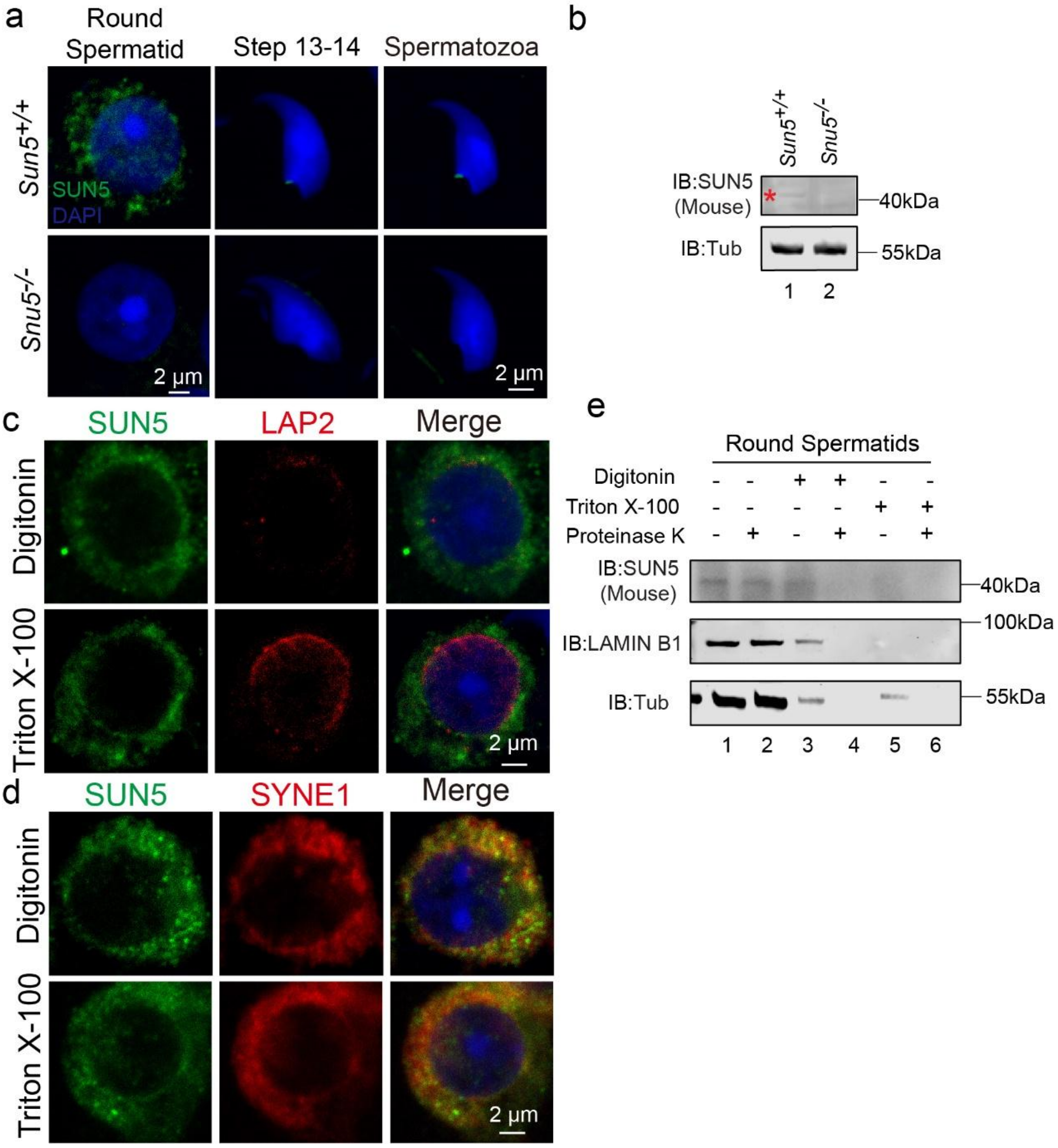

Supplementary Figure 7. The SUN domain of SUN5 is exposed to the cytoplasm of the spermatids. a, The immunofluorescence analysis for SUN5 (green) was performed in *Sun5*$^{+/+}$ and *Sun5*$^{-/-}$ testicular germ cells and mature spermatozoa from caudal epididymis. Nuclei were stained with DAPI (blue). **b**, Immunoblotting of SUN5 was performed in *Sun5*$^{+/+}$ and *Sun5*$^{-/-}$ testes using mouse anti-SUN5 antibody against the SUN5 SUN domain. Tubulin served as a loading control. **c**, The immunofluorescence analysis for SUN5 and LAP2 was performed in testicular germ cells. Cells were treated with either 0.004%

digitonin (on ice) or 0.2% Triton X-100, and then co-labeled with mouse anti-SUN5 antibody against the SUN5 SUN domain (green) and rabbit anti-LAP2 antibody (red). Nuclei were stained with DAPI (blue). **d**, The immunofluorescence analysis for SUN5 and SYNE1 was performed in testicular germ cells. Cells were treated with either 0.004% digitonin (on ice) or 0.2% Triton X-100, and then co-labeled with mouse anti-SUN5 antibody against the SUN5 SUN domain (green) and rabbit anti-SYNE1 (red) antibody. Nuclei were stained with DAPI (blue). **e**, The topology of SUN5 SUN domain in the spermatids. Round spermatids were subjected to in situ proteinase K digestion following differential membrane permeabilization procedures. Immunoblotting of SUN5 were probed with the mouse anti-SUN5 antibody against the SUN5 SUN domain. Permeabilization with digitonin resulted in proteolysis of SUN5 SUN domain, Tubulin and LAMIN B1. The experiment was repeated three times independently with similar results (**a**-**e**).

Supplementary Table1. Primers used in experiments

Primer IDs	Sequence (5' to 3')
Centlein-For	GTAGCTGTGGTGGCATCTCTGGG
Centlein-Rev	TTCTTTATGAAGCGCTGCGTTGG
Centlein-491F	AAGTCAGGCACTTCGGACTC
Centlein-1353R	GCTTTGAGGAAGGAGCCTCT
Centlein-217278R	AAGCAAAGACCTTGACAGCTCCC
