# Supplementary material for: The missing linker between SUN5 and PMFBP1 in sperm head-tail coupling apparatus
Source: Nat Commun. 2021 Aug 13;12:4926. doi: 10.1038/s41467-021-25227-w (PMC8363609; doi:10.1038/s41467-021-25227-w)
Supplement: Supplementary file 3 — Reporting Summary [file 41467_2021_25227_MOESM3_ESM.pdf]

nature research

Corresponding author(s): Li Yuan

Last updated by author(s): Jul 15, 2021

Reporting Summary

Nature Research wishes to improve the reproducibility of the work that we publish. This form provides structure for consistency and transparency in reporting. For further information on Nature Research policies, see our Editorial Policies and the Editorial Policy Checklist.

Statistics

For all statistical analyses, confirm that the following items are present in the figure legend, table legend, main text, or Methods section.

n/a	Confirmed	
☐	☒	The exact sample size (n) for each experimental group/condition, given as a discrete number and unit of measurement
☐	☒	A statement on whether measurements were taken from distinct samples or whether the same sample was measured repeatedly
☐	☒	The statistical test(s) used AND whether they are one- or two-sided<br>*Only common tests should be described solely by name; describe more complex techniques in the Methods section.*
☒	☐	A description of all covariates tested
☒	☐	A description of any assumptions or corrections, such as tests of normality and adjustment for multiple comparisons
☐	☒	A full description of the statistical parameters including central tendency (e.g. means) or other basic estimates (e.g. regression coefficient) AND variation (e.g. standard deviation) or associated estimates of uncertainty (e.g. confidence intervals)
☐	☒	For null hypothesis testing, the test statistic (e.g. F, t, r) with confidence intervals, effect sizes, degrees of freedom and P value noted<br>*Give P values as exact values whenever suitable.*
☒	☐	For Bayesian analysis, information on the choice of priors and Markov chain Monte Carlo settings
☒	☐	For hierarchical and complex designs, identification of the appropriate level for tests and full reporting of outcomes
☒	☐	Estimates of effect sizes (e.g. Cohen's d, Pearson's r), indicating how they were calculated

Our web collection on statistics for biologists contains articles on many of the points above.

Software and code

Policy information about availability of computer code

Data collection	Data were collected using Zeiss ZEN 2.3 software,Leica Application Suite X (LAS X) 3.0 software, Tanon GelCap 5.6 software and UVP TS2 software.
Data analysis	Graphpad Prism 7, ZEN 2.3 lite, LAS X 3.0, Microsoft Excel 2010, Imaris 9.0.2 software.

For manuscripts utilizing custom algorithms or software that are central to the research but not yet described in published literature, software must be made available to editors and reviewers. We strongly encourage code deposition in a community repository (e.g. GitHub). See the Nature Research guidelines for submitting code & software for further information.

Data

Policy information about availability of data

All manuscripts must include a data availability statement. This statement should provide the following information, where applicable:

- Accession codes, unique identifiers, or web links for publicly available datasets
- A list of figures that have associated raw data
- A description of any restrictions on data availability

All data that support the findings of this study are available within the article and its Supplementary Information files or from the corresponding author on reasonable request. Source data are provided with this paper.

Field-specific reporting

Please select the one below that is the best fit for your research. If you are not sure, read the appropriate sections before making your selection.

- [x] Life sciences
- [ ] Behavioural & social sciences
- [ ] Ecological, evolutionary & environmental sciences

For a reference copy of the document with all sections, see nature.com/documents/nr-reporting-summary-flat.pdf

Life sciences study design

All studies must disclose on these points even when the disclosure is negative.

Sample size	No statistical methods were used to predetermine sample size. Mouse experiments were performed at least 3 biologically independent replicates. Moreover, both the experimental and control groups were derived from the same littermates. We followed the conventional way of quantification accepted in many of the published paper in the research field and determined the sample size according to published papers.
Data exclusions	No data was excluded from the results reported here.
Replication	We confirmed the reproducibility of data by independently repeating all of the experiments at least three times. All attempts at replication were successful.
Randomization	Mice were categorized based on their genotypes. The genotypes were determined by PCR. For experiments other than those involving mice, cells used for imaging were selected randomly.
Blinding	Whenever possible, data collection and data analysis were performed by different individuals. Each experiment was designed with proper controls, and samples for comparison were collected and analyzed under the same conditions. The observer unbiasedly and carefully performed the quantification with enough sample number to make sure the conclusion.

Reporting for specific materials, systems and methods

We require information from authors about some types of materials, experimental systems and methods used in many studies. Here, indicate whether each material, system or method listed is relevant to your study. If you are not sure if a list item applies to your research, read the appropriate section before selecting a response.

Materials & experimental systems

n/a	Involved in the study
[ ]	[x] Antibodies
[ ]	[x] Eukaryotic cell lines
[x]	[ ] Palaeontology and archaeology
[ ]	[x] Animals and other organisms
[x]	[ ] Human research participants
[x]	[ ] Clinical data
[x]	[ ] Dual use research of concern

Methods

n/a	Involved in the study
[x]	[ ] ChIP-seq
[x]	[ ] Flow cytometry
[x]	[ ] MRI-based neuroimaging

Antibodies

Antibodies used

The following antibodies were used for immunoblot (IB) and immunofluorescence (IF) studies:
Rat monoclonal anti-CENTLEIN (Clone 9F8), a custom antibody produced by Absea Biotechnology Ltd, IF, 1:20 WB, 1:500
Rabbit anti-SUN5 (17495-1-AP), Proteintech, IF, 1:100
Mouse anti-SUN5 (in-house-generated), IF, 1:100 WB, 1:100
Rabbit anti-PMFBP1 (17061-1-AP), Proteintech, IF, 1:100
Mouse anti-FLAG (M2,F3165), Sigma-Aldrich, WB, 1:2000
Rabbit anti-FLAG (20543-1-AP), Proteintech, WB, 1:2000
Mouse anti-MYC (My3,M192-3), MBL International, WB, 1:2000
Rabbit anti-GFP (50430-2-AP), Proteintech, WB, 1:2000
Mouse anti-MBP (4C6H4, 66003-1-Ig), Proteintech, WB, 1:2000, IF, 1:5000
Mouse anti-GST (12G8, M20007L), Abmart, WB, 1:2000
Mouse anti-GAPDH (1C4, ab1019t), AmeriBiopharma, WB, 1:5000
Mouse anti-γ-tubulin (TU-30, sc-51715), Santa Cruz, IF, 1:200
Rabbit anti-CEP135 (A02C0240), Blue Gene, IF, 1:1500
Mouse anti-Centrin (20H5,04-1624), Millipore, covalently coupled to Alexa Fluor 594, IF, 1:100
GFP-Booster (gba488-100), Chromotek, a gift from Juntao Gao (Tsinghua University), IF, 1:200
Rabbit anti-LAP2 (14651-1-AP),Proteintech, IF, 1:200
Mouse anti-Lamin B1 (3C10G12, 66095-1-Ig), Proteintech,WB, 1:1000

Rabbit anti-SYNE1 (HPA019113), Atlas Antibodies, IF, 1:20
rabbit anti-α-Tubulin (AC007), ABclonal, WB, 1:2000.
Following secondary antibodies were used:
HRP- conjugated goat anti-mouse IgG (ZB-2305), Zhong Shan Jin Qiao, WB, 1:4000
HRP- conjugated goat anti-rabbit IgG (ZB-2301), Zhong Shan Jin Qiao, WB, 1:4000
Goat anti-rabbit FITC (ZF-0311), Zhong Shan Jin Qiao,IF,1:200
Goat anti-rabbit TRITC (ZF-0316), Zhong Shan Jin Qiao, F,1:200
Goat anti-mouse TRITC (ZF-0313),Zhong Shan Jin Qiao,IF,1:200
Donkey anti-rabbit Cy5 (711-175-152), Jackson ImmunoResearch,IF,1:200
Alexa Fluor 594 goat anti-rat IgG (A11007), Invitrogen,IF,1:1500
Alexa Fluor 488 goat anti-rat IgG (A11006), Invitrogen,IF,1:1500

Validation

The newly generated antibodies in this study were validated by western blotting and immunostaining using WT and knockout mouse controls. The following antibodies were validated for immunoblot (IB) and immunofluorescence (IF) in our previous studies:rat anti-CENTLEIN (clone 9F8, IF, 1:20; WB, 1:500), mouse anti-SUN5 (IF, 1:100; WB,1:100). Other antibodies were purchased from commercial suppliers and validated by the vendor.
SUN5 (17495-1-AP), Species Reactivity: human, mouse; Applications: IF, WB, ELISA
PMFBP1 (17061-1-AP), Species Reactivity: human, mouse, rat; Applications: WB, ELISA, IF
FLAG (M2,F3165), Species Reactivity: all; Applications: WB
FLAG (20543-1-AP), Species Reactivity: recombinant protein; Applications: IF, WB, ELISA
MYC (My3,M192-3), Species Reactivity: Myc-fusion proteins; Applications: IP, WB, ICC, FC
GFP (50430-2-AP), Species Reactivity: aequorea victoria, recombinant protein; Applications: IF, IP, WB, ELISA
MBP (4C6H4, 66003-1-Ig), Species Reactivity: recombinant protein; Applications: IF, IP, WB, ELISA
GST (12G8, M20007L), Species Reactivity: all; Applications: IF, WB, IP, ELISA
GAPDH (1C4, ab1019t), Species Reactivity: human, mouse, rat; Applications: WB
γ-tubulin (U-30, sc-51715), Species Reactivity: human, mouse, rat; Applications: WB, IP, IF
CEP135 (A02C0240), Species Reactivity: human, mouse; Applications: WB, ELISA
Centrin (20H5,04-1624), Species Reactivity: human, mouse, rat; Applications: WB, IP, ICC
GFP-Booster (gba488-100), Species Reactivity: GFP-fusion proteins; Applications: IF
LAP2 (14651-1-AP), Species Reactivity: human, mouse, rat; Applications: IF, IHC, IP, WB,ELISA
Lamin B1 (3C10G12, 66095-1-Ig), Species Reactivity: human, mouse, rat; Applications: FC, IF, IHC, IP, WB,ELISA
SYNE1 (HPA019113), Species Reactivity: human; Applications: IF, IHC
α-Tubulin (AC007), Species Reactivity: human, mouse, rat; Applications: WB, IF
CEP135 (A02C0240) and Centrin (20H5,04-1624) antibodies can be used for IF according to published papers (Fang, G. et al. Journal of Cell Science, 2014; Fishman, E. L. et al. Nature Communications, 2018). SYNE1 (HPA019113) can recognize the mouse SYNE1 according to published paper (Gob, E. PLoS One, 2010).
We further validated the antibodies by staining positive and negative cell lines/tissues using protocols recommended manufactory. The specificity was further determined by evaluating the morphology of stained cells in immunostaining assay and the size of detected band in immunoblotting assay. All antibodies were initially tested against unstained controls and the antibody dilution ratio was optimized for each antibody.

Eukaryotic cell lines

Policy information about cell lines

Cell line source(s)	HEK293T cells were purchased from ATCC.
Authentication	The HEK293T cell line was authenticated by Short Tandem Repeat (STR) profiling analysis.
Mycoplasma contamination	HEK293T cells were tested negative for mycoplasma contamination.
Commonly misidentified lines (See ICLAC register)	No commonly misidentified cell lines were used.

Animals and other organisms

Policy information about studies involving animals; ARRIVE guidelines recommended for reporting animal research

Laboratory animals	Male 8-week-old Centlein+/-, Centlein-/-, Sun5-/-, Pmfbp1-/- mice and littermate controls were used for this study. Mice were housed in the same animal facility on a 12-h reverse light/dark cycle. The animal facility was maintained at a temperature of 22-24°C with 40–60% humidity.
Wild animals	This study did not involve wild animals.
Field-collected samples	This study did not involve samples collected from the field.
Ethics oversight	Animal experiments were conducted under the protocol and approval (IOZ20170079) of the Animal Care and Use Committee of the Institute of Zoology, Chinese Academy of Sciences, China. The present study is compliant with all relevant ethical regulations regarding animal research.

Note that full information on the approval of the study protocol must also be provided in the manuscript.
